# Stem-like features of cancer cells on their way to metastasis

**DOI:** 10.1186/s13062-016-0135-4

**Published:** 2016-07-26

**Authors:** Sofia Gkountela, Nicola Aceto

**Affiliations:** Department of Biomedicine, Cancer Metastasis, University of Basel, Mattenstrasse 28, CH-4058 Basel, Switzerland

**Keywords:** Cancer metastasis, Circulating tumor cells, CTCs, CTC clusters, Stem-like features

## Abstract

**Abstract:**

More than 90 % of cancer-related deaths are due to the development of a systemic metastatic disease. Clearly, much remains to be understood about the biological principles that govern human cancer metastasis, aiming at the ambitious objective to decrease metastasis-related mortality in patients. For many years, research on metastasis has been conducted in great part on experimental mouse models, mainly because of the difficulties in sampling, longitudinal studies, and molecular interrogation of a human metastatic disease. However, recently, extraordinary advances in microfluidic technologies are allowing the isolation and characterization of human circulating tumor cells (CTCs) that escaped a primary tumor mass and are in the process of seeding a distant metastasis. Analysis of human CTCs has now revealed important features of cancer metastasis, such as the high metastatic potential of CTC-clusters compared to single CTCs, the dynamic expression of epithelial and mesenchymal markers on CTCs during treatment, and the possibility to culture CTCs from patients for a real-time and individualized testing of drug susceptibility. Nevertheless, several aspects of CTC biology remain unsolved, such as the characterization of the stem-like cell population among human CTCs. Here, we focus on describing the latest findings in the CTC field, and discuss them in the context of cancer stem cell biology. Defining the molecular features of those few metastasis-initiating, stem-like CTCs holds the exceptional promise to develop metastasis-tailored therapies for patients with cancer.

**Reviewers:**

This article was reviewed by Elisa Cimetta, Luca Pellegrini and Sirio Dupont (nominated by LP).

## Background

Cancer is the outcome of a complex, multi-step evolutionary process during which normal cells acquire aberrant features that enable them to become tumorigenic and ultimately malignant [1]. Currently, despite remarkable advances in diagnosis, surgical techniques and anti-cancer therapies, cancer remains among the leading causes of death worldwide, with a projected 70 % increase in new cases in the next 20 years according to the World Health Organization (WHO). It is estimated that, annually, more than 8 million people die from cancer worldwide, and more than 90 % of these cancer-related deaths are due to the formation and progression of a systemic metastatic disease [2–6]. These numbers reflect our limited understanding of the key processes that drive human cancer metastasis. To date, most of our understanding of the metastatic process derives from experimental mouse models. These models are well established, can be genetically manipulated and are ideal for studying tumorigenic and metastatic properties of human cancer cell lines, as well as genetically-engineered endogenous mouse cancer cells. However, although instructive, conclusions derived from mouse models need to be validated in human specimens. This has proven to be extremely challenging mainly because human metastatic sites are difficult to biopsy, and the limited material obtained is often highly admixed with surrounding healthy stromal tissue. On the other hand, recent breakthrough technological advances that are emerging, are now enabling the isolation and characterization of human cancer cells in transit through the bloodstream, on their way to distant organs, with an outstanding potential to reveal those fundamental events that underlie the development of human cancer metastasis [7, 8].

### Circulating Tumor Cells (CTCs)

Circulating tumor cells (CTCs) represent cancer cells that detach from a solid tumor mass and, using the blood circulatory system, are in the process of colonizing distant organs to give rise to metastasis [1]. CTCs have been detected in the majority of cancers of epithelial origin, including breast, colon, prostate, and pancreas but also in cancers of non-epithelial origin, such as glioblastoma multiforme and melanoma [7–12]. Most importantly, their presence has been correlated with a poor prognosis in several major epithelial cancer types, suggesting that CTCs contribute actively to the metastatic process [7, 13, 14]. However, in patients, CTCs are extraordinarily rare compared to normal blood cells (approximately one CTC among a billion normal blood cells), and their isolation and molecular characterization has been hampered for a long time by technological constraints. Furthermore, variable expression levels of cancer-associated cellular markers represent a major challenge in identifying these rare cells in a viable state, among a large number of blood cells, highlighting the need for specialized technologies for their isolation and characterization.

In the recent years, several platforms have been established that take into account both cell surface expression patterns as well as physical or functional properties of CTCs for their isolation [9, 15–18]. For epithelial cancer types, antigen-dependent isolation devices rely mostly on the expression of cell surface Epithelial Cell Adhesion Molecule (EpCAM) to isolate CTCs, given its high expression in most of the tumor cells of epithelial origin [19]. For example, the only FDA-approved platform for CTC enumeration is the CellSearch system, which uses a two-step procedure to isolate CTCs [17, 18]. In the first step, plasma components are removed by centrifugation, while EpCAM-positive CTCs are captured using immunomagnetic beads. In the second step, CTCs are permeabilized and stained for the expression of additional markers, such as the epithelial cytokeratins (CK) 8, 18, and 19. Meanwhile, contaminating leucocytes are identified with a staining against CD45, a widely expressed leukocyte marker. At the end of the procedure, captured cells are placed on an integrated analyzer for scanning and CTCs are identified as being positive for the expression of CKs and DAPI, while being negative for CD45. Even though CellSearch is currently considered as the gold standard in capturing CTCs in a clinical setting, a disadvantage of this technology is that ultimately isolated CTCs are non-viable due to fixation and additionally, the antigen-dependent approach for CTC-isolation may in fact favor the enrichment of CTCs with higher EpCAM expression, over CTCs with lower EpCAM levels as a consequence of cancer heterogeneity [20], dynamic protein turnover [21], or the down-regulation of epithelial markers in favor of mesenchymal-like traits, in a process known as Epithelial-to-Mesenchymal Transition (EMT) [8, 22–24]. As a consequence, new emerging technologies tend to focus on antigen-independent methods for CTC isolation, yet they are designed based on different principles. For example, isolation of CTCs independently of the expression of tumor-associated antigens has been achieved via size-based depletion of normal blood cells, leveraging on the notion that CTCs are slightly bigger than red or white blood cells (approximately 12-25 μm for a single CTC, vs 8 μm and 7-15 μm, for red and white blood cells respectively) [25, 26]. Filter-based platforms such as Parsortix and ScreenCell capture larger CTCs, while smaller white blood cells (WBCs) and blood components like platelets and red blood cells (RBCs) flow through the microfluidic channels [27, 28]. Alternatively, the spiral biochip is another newly developed ultra fast, size-based microfluidic device that can isolate larger CTCs from patient blood samples within a few minutes. This chip relies on a combination of hydrodynamic forces that are generated when blood flows through spiral microchannels. These forces position larger CTCs towards the inner walls of the spiral channel, while smaller blood components are pushed towards the opposite outer wall [29]. However, further studies need to be performed with this technology to assess whether the higher shear forces, compared to those that are normally present in physiological blood circulation, could influence some of the properties of CTCs. On the other hand, the CTC-iChip combines an initial hydrodynamic cell separation of nucleated cells, including CTCs and WBCs, from other blood components, followed by an immunomagnetic depletion of antibody-tagged WBCs [7]. This approach, similarly to the other size-based approaches mentioned above, results in the antigen-independent enrichment of CTCs from whole blood samples, allowing their molecular characterization.

Antigen-independent isolation of CTCs from cancer patients is key to understanding the biology of cancer metastasis in a variety of cancer types, including those of epithelial but also non-epithelial origin. Recently, using the CTC-iChip and the Herringbone ^HB^CTC-chip microfluidic platforms, as well as density gradient centrifugation, CTCs were isolated from the blood of breast, prostate and pancreatic cancer patients [7, 30, 31], as well as patients with small cell lung cancer, glioblastoma multiforme and melanoma [32–34]. Downstream, single cell resolution transcriptional profiling of these cancer cells that are *en route* to metastasis has revealed a great degree of heterogeneity among them within the same patient, but also among CTCs from different patients. Interestingly, these studies revealed a role for non-canonical WNT signaling in drug resistance and establishment of metastases in pancreatic and prostate cancer patients [30, 31]. In human breast CTCs, a dynamic expression of epithelial versus mesenchymal markers in response to treatment was observed using quantitative RNA-*in situ* hybridization, demonstrating for the first time a mesenchymal-like phenotype in human metastatic cells [8]. Similarly, in glioblastoma multiforme, mesenchymal markers were enriched in CTCs over neural differentiation markers [33]. In small cell lung cancer, CTCs were shown to be tumorigenic upon transplantation in immunocompromised mice and more importantly, the xenograft tumors matched those morphological and genetic features of the primary tumor in the patient of origin, and were predictive of treatment response [32].

All together, recent technological breakthroughs are allowing us to gain fundamental insights into CTC heterogeneity in different types of cancers and patients. However, it is very important to highlight that in any given tumor type, the number of CTCs present in the bloodstream appears to largely exceed the number of clinically detectable metastatic foci, indicating that most CTCs will not lead to metastasis, and that only very few will have those features that will enable them to seed a metastatic disease.

### CTC clusters

The identification and characterization of the subset of metastasis-initiating cells among the CTC population in patients is of paramount clinical importance. The majority of CTCs circulate in the blood of cancer patients as single cells, however they can also be found as clusters of 2-50 cells, with the ratio of single vs clustered CTCs varying significantly among different patients, and along disease progression [7, 30, 31]. While the role of CTC clusters in the metastatic process remained unknown for a long period, recently, their presence in the blood circulation of patients with metastatic breast, lung or prostate cancer was correlated with poor metastasis-free survival and overall survival, suggesting that CTC clusters are key players in the spread of cancer cells to distant metastatic sites [7, 35, 36]. By using the CTC-iChip technology in combination with a micromanipulator, both single CTCs and CTC clusters from patients with metastatic breast cancer were recently isolated and subjected to RNA sequencing profiling [7]. Data analysis revealed that CTC clusters upregulate a set of genes that include the cell-cell junction component plakoglobin. In breast cancer patients, increased expression of plakoglobin in the primary tumor is indicative of a decreased metastasis-free survival, while in mouse xenograft models, knockdown of plakoglobin expression in orthotopic mammary tumors suppresses spontaneous CTC cluster formation and lung metastases [7].

In the same study, using two independent mammary tumor mouse models, it was shown that CTC clusters are oligoclonal in origin and do not arise from the aggregation or proliferation of single CTCs within the circulatory system [7, 8]. Rather, CTC clusters arise when a group of malignant cells detaches from a solid tumor deposit and enters into the blood circulation. By color-coding primary tumor cells to distinguish single versus clustered CTCs, it was also shown that even though single CTCs are more frequent in blood circulation, lung metastases arise preferentially from CTC clusters. In fact, CTC clusters were found to be up to 50 times more likely to form lung metastatic deposits compared to single CTCs in this experimental setup. Using in vivo flow cytometry in mouse models to assess circulation half-life of single and clustered CTCs, it was found that larger CTC clusters are rapidly cleared (usually within 10 min) from the blood circulation, due to their entrapment in smaller capillaries at distant organs [7]. Thus, compared to the 30 min half-life of single CTCs in blood circulation, it is possible that current blood sampling techniques underestimate the number of CTC clusters released in circulation due to their shorter half-life. However, the rapid clearance of CTC clusters at distant organs indicates that clusters reach potential metastatic sites at a higher rate compared to single CTCs. Whether the higher metastatic propensity of CTC clusters correlates with the faster rate at which they reach distant organs in patients, or whether this indicates that metastasis-initiating cells with stem-like properties are preferentially enriched in clusters versus single CTCs is currently unknown.

### Stem-like properties of cancer cells

By definition, stem cells are cells that are resident in virtually all normal tissues and have the ability, upon cellular division, to both self-renew and give rise to cells that will differentiate into more specialized cell types [37, 38]. As a consequence to this definition, in cancer, the so called “cancer stem cells” are defined as those cells with stem-like properties, which can self-renew and differentiate, resulting into a population of cells that has the long-term ability to clonally reproduce the tumor itself and its inherent heterogeneity [39, 40]. Functional repopulation assays are currently the gold standard for measuring stem-like properties of cells. For cancer biology, serial transplantations in mice or *in situ* tracking are used to ensure that cancer cells with stem-like properties have the long-term ability to clonally maintain the tumor and regenerate the heterogeneous tumor mass. These assays are designed in order to simultaneously assess the self-renewal and differentiation potential of putative stem-like cancer cells. The principle for the serial transplantation assay is that cancer stem-like cells must have the ability to re-establish the primary tumor, while maintaining both heterogeneity and self-renewing ability upon sequential transplantations into animal models [41]. During *in situ* clonal assays, a population of cells is initially labeled, most commonly by the addition of a fluorescent marker, to allow long-term tracing of the progeny in the recipient mouse, in order to assay again for self-renewal and differentiation potential [42].

However, one of the major challenges in these studies is to identify those expression markers that faithfully define a cancer stem cell phenotype. Undeniably, there is a great degree of heterogeneity between stem-like cancer cells themselves and among different tumor types [37, 38], arguing that the identification of faithful stem-like markers in cancer cells is an extraordinarily difficult task. The identification of a small population of cells enriched with a tumor-initiating potential was first reported in Acute Myeloid Leukemia (AML) and it was based on the expression pattern of cell adhesion markers CD34 and CD38 [43]. Using flow-sorting techniques it was shown that a fraction of CD34^+^/CD38^-^ but not CD34^+^/CD38^+^ cells were able to initiate AML upon serial transplantations in immunodeficient mice. These studies provided proof that not all tumor cells are equal in their tumor-initiating potential and paved the way for the identification, based on expression of cell surface markers, of similar populations with stem-like properties in other solid tumors. For instance, in brain and lung cancer, the expression of CD133, a transmembrane protein with poorly understood signaling function, correlates with tumor initiating potential upon serial transplantations in immunodeficient mice [44, 45]. Similarly, in colon cancer as well as in ovarian tumors, cancer-initiating cells are highly enriched in the CD133 expressing fraction [46–48]. In pancreatic cancer, combination of two distinct expression patterns, such as of CD44/CD24 together with the epithelial-specific antigen (ESA) or CD133/CXCR4, can identify the cancer stem-like population [49, 50]. However, CD133 expression combined with that of CXCR4, a mediator of cell migration, was also shown to enrich for a population that in addition to stem-like features, also displayed a metastatic potential in pancreatic cancer [50]. In breast and prostate cancer in particular, the subpopulation of cancer cells with stem-like properties is identified as being enriched in the CD44^+^/CD24^-^ fraction [51, 52]. In breast cancer, combination of CD44 expression with that of alcohol dehydrogenase (ALDH), which is also expressed in hematopoietic progenitor cells, could further refine the tumorigenic population [53, 54]. ALDH alone was also used as an expression marker in both breast and prostate cancer to identify a distinct population of stem-like cells in addition to the CD44^+^/CD24^-^ fraction [55, 56].

Taken together, these studies indicate that combinations of different cell surface expression markers have been used to identify distinct tumor-initiating populations in solid tumors, but that use of these expression markers may not yet be sufficiently refined to identify unequivocal stem-like populations in different human cancers, especially considering the high degree of mutational and cellular heterogeneity of human tumors in the metastatic setting, and upon resistance to therapeutic agents [57]. Further, it has been shown that the percentage of putative stem-like cancer cells in each model may vary dramatically, depending on experimental conditions. Factors such as the use of different recipient mouse strains, as well as assaying tumors at different stage of progression and treatment may explain some of the discrepancies between those studies aimed at assessing the stem-like population in solid tumors (Reviewed in [57]). Univocally though, the studies summarized above point to the fact that, in order to dissect the heterogeneity within the putative stem-like cancer populations, those functional assays that are currently used to assess stemness such as the serial transplantation assay, will need to be redesigned in order to assay for both self-renewal and differentiation potential in clonal populations arising from single cells. Tracing both self-renewal, differentiation and tumorigenic potential to a single cell level will also be crucial for identifying true tumor-initiating cells endowed with stem-like potential in cancer, and discriminating them from subpopulations of cells that are only enriched in proliferation or survival properties but lack true stem-like potential. To this regard, there is also a need to reassess culture methods in vitro, and most importantly ensure that clonal populations can arise from single cells in vitro without the need to exogenously supplement saturating amounts of growth factors. Altogether, these studies point towards the need to refine both the use of expression markers and related functional assays, especially considering the possibility that is now available, to interrogate cancer cells at higher resolution and at the single cell level [58, 59].

### Stem-like properties of cancer cells in circulation

Expression of cancer stem-like markers in various cancer types has been shown to correlate with the occurrence of metastasis and reduced survival in patients, supporting the hypothesis that cancer cells enriched in stem-like features are the precursors of metastasis (reviewed in [57, 60]). Recent single cell-resolution RNA sequencing data obtained from CTCs of patients with prostate and breast cancer may help gain insights into the expression of stem-like markers, specifically in cancer cells as they transit through the bloodstream, as CTCs isolated from these cancer patients were shown to express *CD44*. Moreover, 60 % of the CTCs profiled from prostate cancer patients were also enriched for *ALDH7A1* and other putative stem-like markers such as *KLF4* [7, 31]. However, the expression dynamics and the heterogeneity regarding the expression of these markers have not yet been quantitatively assessed in CTCs. Most importantly, it will be necessary to link the expression patterns of these putative stem-cell markers in CTCs with enhanced tumor-initiating and metastatic potential. To date, even though CTCs isolated from patients are able to form tumors in mice, they have not been assessed for their ability to long-term self-renew and regenerate a highly heterogeneous cancer upon serial transplantations [7, 30]. Arguably, additional functional assays should be implemented, that can assess the stem-like properties of cancer cells at the single cell level, and will enable the correlation of stem-like marker expression with the metastatic potential of putative stem-like CTCs.

**Fig. 1:**
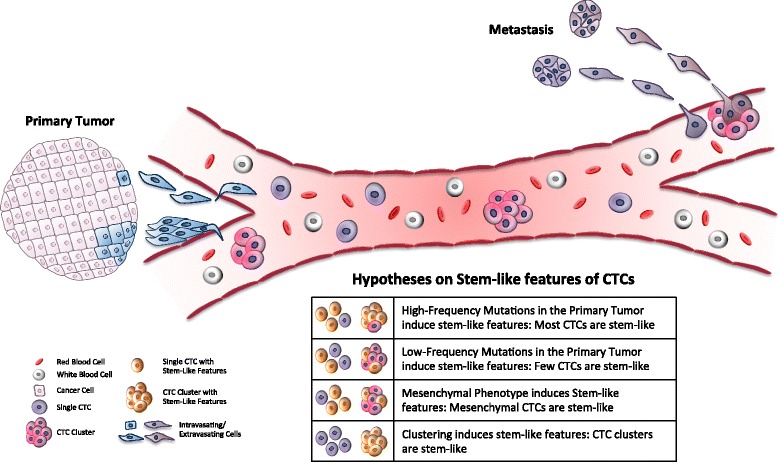
Stem-like features of CTCs. Schematic representation of different hypotheses describing how single CTCs or CTC-clusters could acquire stem-like features

On the other hand, it is increasingly evident that stem-like properties may represent an extraordinary advantage for CTCs as they encounter a number of challenges during the metastatic process. For example, a first major challenge that CTCs face, is the need to suppress anoikis, a form of programmed cell death that is triggered in epithelial cells upon loss of adhesion to surrounding cells. Once in blood circulation, CTCs are striving to survive in a hostile, immune cells-rich environment while they are subjected to high shear forces that challenge their structural integrity. Additionally, if successful, CTCs that reach distant organs will need to adapt to the foreign environment in order to seed a metastatic lesion. In this context, it is not surprising that the majority of molecular pathways that regulate self-renewal, such as PI3K/AKT, PTEN, JAK/STAT, seem to be also essential for cancer onset and metastasis, while increasing survival signals that provide resistance to cell death and chemotherapeutic agents [61–64]. Whether stem-like properties are a feature of some CTCs and whether they are enriched in single versus clustered CTCs remains an open question. The fact that CTC clusters are more prone to establishing metastases compared to single CTCs in mouse xenograft models could indicate that stem-like properties are enriched in CTC clusters over single CTCs. On the other hand, it is also possible that stem-like properties are equally enriched in both single CTCs and CTC clusters, but the higher metastatic propensity observed with CTC clusters is reflective of their physical advantages, such as the greater likelihood to be trapped in small capillaries at distant sites. To this regard, the presence of cell-cell junctions in CTC clusters could help overcome anoikis in the bloodstream and provide survival signals along the metastatic process, including during the early dissemination to distant organs. The actual contribution of specific mutations and deregulated signaling pathways, mesenchymal-like features and CTC-clustering for the generation of metastasis-initiating cells remains to be addressed (Fig. 1).

Another important hint connecting stem-like properties with CTCs is the protocol that has been implemented for CTC cultures [65]. After testing a number of different culture conditions it was shown that CTCs could be propagated in vitro only if they were cultured in hypoxic conditions, as tumor spheres in serum-free media supplemented with epidermal growth factor (EGF), B27 and basic fibroblast growth factor (FGF2). Interestingly, given the fact that CTCs have a limited half-life in blood circulation, the requirement for hypoxic conditions for their propagation could reflect the oxygen levels within the tumor tissue of origin, rather than the oxygen concentration within the arterial or venous circulation. Similarly to CTCs, culture of human embryonic stem cells (hESCs) under hypoxic conditions is also important for regulating pluripotency and proliferation of these cells in vitro [66]. Additionally, for hESC and human induced pluripotent cell (hIPSC) culture in vitro, FGF2 is required for promoting self-renewal and survival in several different ways, such as via the activation of the PI3K/AKT and MAPK signaling pathways [67], but also through promotion of cell adhesion, highlighting the importance of cell-cell contact in maintenance of self-renewal and survival in hESCs [68]. To this regard, hESCs can be propagated in an undifferentiated state in vitro essentially indefinitely by forming rather tightly packed colonies of individual cells that are connected through various complexes, such as adherence junctions, tight junctions, desmosomes and gap junctions [69]. Undoubtedly, not all stem cells require FGF2 signaling for maintaining pluripotency in vitro. In fact, mouse ESCs that are functionally very similar to hESCs, are destabilized by FGF2 and depend on the activation of the LIF/Stat3 signaling pathway [70]. However, as opposed to conventional hESCs that display very poor clonal efficiency and survival when dissociated to single cells, mESCs not only tolerate single cell dissociation but also manage to maintain pluripotency under these conditions [71]. Therefore, we speculate that in systems such as hESCs that depend on FGF2, the link between pluripotency and cell-cell adhesion could support a model in which CTC clusters are enriched in stem-like properties. Further, not only cell-cell adhesion but additionally other signaling molecules could be promoting stemness in CTCs [30, 31, 72].

Recent molecular studies on CTC clusters indicate that there is a great degree of cellular and molecular heterogeneity among different CTC clusters, even among those profiled from the same patient [7, 30, 31]. This heterogeneity unquestionably also reflects differences in expression profiles of individual CTCs that form the same cluster and potentially also reflects the expression of stem-like features. Therefore, it is possible that only a few cells within a CTC cluster are in fact retaining stem-like properties and are able to form metastases, while other surrounding cells display reduced or no stem-like characteristics and do not contribute to the metastatic process. Additionally, other, non-cancerous cell types, such as macrophages, platelets and even fibroblasts and epithelial cells, were shown to bind CTC clusters [7, 73–75]. These interactions can affect the molecular profile of the cells they are in direct contact with, further promoting heterogeneity and enhancing the overall fitness of CTCs. For example, platelets that bind to CTCs may release transforming growth factor β (TGF-β), which in turn can provide additional survival signals [75]. Moreover, platelets have also been shown to induce EMT, a transcriptional program which is orchestrated by a number of embryonic transcription factors, that was also suggested to promote the expression of stem-like markers, conferring malignant traits such as invasiveness and resistance to apoptosis [22, 76]. Alternatively, CTC-associated platelets can release adenine nucleotides and facilitate extravasation by inducing an opening at the endothelial barrier [77], while macrophages that bind to CTCs can promote tissue remodelling and help extravasation through capillary walls in the lung [78]. Moreover, tumor-derived fibroblasts or endothelial cells that bind to CTC clusters could act as passenger stromal cells that form part of the tumor microenvironment at the distant site, therefore facilitating the initial growth of CTCs and ultimately promoting metastasis [74, 75].

Therefore, it is evident that in order to dissect the heterogeneity that is present in CTCs, additional studies that aim at profiling these cells at single cell resolution are necessary. In respect to identifying stem-like features in CTCs, it will be important to first identify robust expression markers that faithfully report the stem-like potential of these cells, as well as appropriate functional assays that assess their self-renewal and differentiation potential while also evaluating their metastatic propensity.

## Conclusions and open questions

One of the most important challenges for cancer stem cell biology today represents the identification of those markers that robustly define cancer stem cells within a highly heterogeneous human tumor, and along the metastatic cascade. With regard to metastasis, it will be interesting to identify whether markers that confer stem-like properties are specifically enriched in a subset of CTCs. Even more importantly, mechanistic studies will need to address the direct molecular mechanisms by which these markers contribute to the metastatic cascade. Undoubtedly, there is also an increasing need to refine those functional assays currently employed for assessing stem-like as well as tumorigenic potential in cancer, so that cancer cells are interrogated at single cell resolution. This is a crucial step to enable the identification of *bona fide* stem-like cancer cells among a highly heterogeneous cell population. Since cancer is manifested on a genetically diverse population of cells, it will also be important to identify the mutational events that are associated with enhanced stem-like properties of cancer cells. To this regard, it was recently shown that mutations in the PI3K pathway have the ability to induce multipotency in breast cancer [79, 80]. Whether other mutations, and more importantly whether low frequency mutations that can be easily overlooked at the time of diagnosis, have the ability to fine-tune malignant properties of cancer cells by building upon an already tumorigenic program, enhancing stem-like features and becoming predominant during disease progression, remains an open question (Fig. 1).

Moreover, the contribution of non-genetic elements still needs to be addressed, and in particular the potential role of DNA methylation, chromatin structure and other epigenetic modifications in the metastatic process. Can certain genetic or epigenetic signatures promote stem-like features and are these signatures enriched in a subset of CTCs? Further, how do stromal cells relate to multipotency and do they act by promoting and/or maintaining stem-like features in CTC clusters?

Another interesting aspect of CTC biology is the potential link with anti-cancer immunotheraphy strategies. For example, CTCs isolated from breast cancer patients with advanced metastatic disease have been shown to express Programmed Death Ligand 1 (PD-L1) in order to avoid immune system detection by inhibiting T cell-mediated immune response [81]. The PD1/PD-L1 interaction has been the basis for the development of a number of immunotherapies in patients with several solid tumors. In such cases, treatment with anti-PD-L1 blocking antibody has shown very promising clinical responses in a subset of patients [82]. Similarly, in melanoma, PD-1 as well as Cytotoxic T lymphocyte antigen-4 (CTLA-4), another key negative regulator of T cell activation, have been exploited towards the development of immune checkpoint inhibitors [83–87], although at present it is very hard to predict which patients will benefit from this therapeutic strategy. In this context, CTC analysis may help to stratify patients and to identify those who would benefit from an immunotherapy approach.

Clinically, a fundamental question is whether we can learn how to target the metastasis-initiating cell population within heterogeneous tumors. To date, targeted cancer therapies have not been successful in preventing the development of metastases in cancer patients, highlighting an enormous need for metastasis-tailored drug discovery programs. Along this line, the isolation and detailed molecular profiling of CTCs represents an outstanding opportunity to define the molecular, as well as the stem-like features of metastasis-initiating cells, contributing to advancing our knowledge on the biology of metastasis in patients with cancer.

## Reviewers’ comments

### Reviewers’ report 1: Elisa Cimetta, Columbia University, New York, United States of America

#### Revision 1

Summary: The authors satisfactorily addressed all of the reviewers’ comments. The manuscript will be a great stimulus for the field of CTCs research, clearly reviewing the current scientific advancements, discussing the criticalities and posing relevant open questions.

Authors’ response: *We thank the Reviewer for the appreciation of our manuscript*.

Minor issues: Page6, line 44: “is that, cancer” should be “is that cancer”. Page7, line 26: “has however also be used” should be “was also used”.

Authors’ response: *We thank the Reviewer and have updated the text accordingly*.

### Original comments

Summary: The authors are presenting a comprehensive review of an extremely relevant issue in the field of Cancer Cells biology. The focus is on human circulating tumor cells (CTCs) and on the understanding of the biological principles that govern human cancer metastasis. The manuscript is well written, well thought and, most importantly, gives the reader abundant food for thought. There is a clear illustration of the state of the art and the associated main criticalities. I particularly appreciated the open questions in the Conclusions section, as they highlight areas of interest where new hypothesis and further research could lead to great advances in the field.

Authors’ response: *We thank the Reviewer for the positive comments on our manuscript*.

Recommendations:

1. There are no serious deficiencies in the manuscript. Two minor observations follow: With reference to the spiral biochip for CTCs separation (pg.4, from line 23 on): could the authors comment on the potential effects (membrane damages, pathways activation, etc) elicited by shear forces on the exposed cells?
  Authors’ response: *In regard to the spiral biochip, we have now addressed this point in the text. We indicate that further studies will be needed to determine a potential effect of the higher shear forces, used by the spiral biochip, on the properties of CTCs*.
2. The authors state that “..cell-cell adhesion in CTCs is essential for promoting stem-like features..” (pg.8, from line 41 on): could this be linked to the fact that CTCs clusters appear to be enriched in stem-like cells?
  Authors’ response: *We agree with the speculation raised by the Reviewer. This point is now addressed in greater detail in the text*.

Minor issues: Page8, line 58: “we shown” should maybe be “were shown”?

Authors’ response: *We thank the Reviewer for noticing the typo. This has now been corrected in the text*.

### Reviewers’ report 2: Luca Pellegrini, Université Laval, Canada

#### Original comments

Summary: The review addresses how single and clustered tumor cells that circulate in the bloodstream of cancer patients (CTCs) contribute to metastasis formation. It provides an overview of the methods used to isolate CTCs and how they have been used to understand the origin, fate, molecular fingerprint, and malignancy of this type of cell. An extensive part of the review is dedicated in putting together a case for considering CTCs as stem-like cells, a possibility with important implications for the search of antimetastatic pharmacological approaches. I found it a very well written and highly informative work from a junior faculty that has already contributed outstanding work in the CTC field: readers of Biology Direct that are in different fields of cell biology should find the review useful and educative; and CTC experts should appreciate its vision and sharpness in identifying the challenges ahead and the outstanding questions that remain to be addressed.

Authors’ response: *We thank the Reviewer for his very positive comments on our review*.

Recommendations:

1. I would recommend to expand the chapter “conclusions and open questions”: some clever and important ones are scattered throughout the text; so, it would be best to repeat them here.
  Authors’ response: *We thank the Reviewer for his suggestion. We have expanded this section in our revised manuscript to better describe the need to identify reliable stemness-associated markers in cancer, especially at a single cell resolution. We also highlight the need to refine the functional assays currently employed for assessing stem-like and tumorigenic potential in cancer cells*.
2. I would also recommend to address the link between CTC and anti-cancer immunotherapy.
  Authors’ response: *We have also addressed this point in the section “conclusions and open questions”, giving special emphasis to the PD-L1/PD1 interaction and the fact that, while very promising, it is at present hard to predict which patients will benefit from an immunotherapy approach. In the future, the analysis of CTCs may serve a tool to stratify patients in this regard, and to identify those that are more likely to benefit from such therapy*.

Minor issues:

1. Define and explain EpCAM
  Authors’ response: *We have adapted the text and now provide a better definition and explanation of the choice of EpCAM as a marker for epithelial CTC isolation*.
2. Explain the Cell Search system for the readers that are not familiar with it.
  Authors’ response: *Following the reviewer’s suggestion, we have now expanded the part referring to the CellSearch system and explained it in greater detail.*
3. Page 4, line 18: clarify if this is the size of single or clustered CTC?
  Authors’ response: *We have clarified this point and thank the Reviewer for noticing this omission*.
4. Page 5, line 17-18: specify the relative percentage (or ratio) of single vs. clustered CTC.
  Authors’ response: *We now provide a statement on the ratio of single vs clustered CTCs, yet specifying that it can vary dramatically among different patients and along disease progression*.
5. Page 8, line 44-46: this sentence is not clear.
  Authors’ response: *We have now reworded and clarified the sentence*.
6. Page 8, line 49: explain the type of heterogeneity.
  Authors’ response: *We now state that CTC clusters have a high degree of both cellular and molecular heterogeneity*.
7. Page 8, line 58: correct “we shown”.
  Authors’ response: *We have corrected this in the text, and thank the Reviewer for noticing the typo*.
8. Do not abbreviate/use acronyms for things that are mentioned only twice (e.g. EMT).
  Authors’ response: *We have followed this suggestion and corrected it throughout the text*.

### Reviewers’ report 3: Sirio Dupont, University of Padova, Italy (Nominated by Luca Pellegrini, University of Cambridge, United Kingdom)

#### Revision 1

Recommendations:

1. On my point 7, Page 9, around line 40. The authors added in this revised version the requirement for Wnt in the maintanance of hESC pluripotency, and make a parallel with CTC culture conditions. However, the role of Wnt in hESC is highly controversial in the field, as many found evidence that Wnt actually promotes hESC differentiation, recapitulating the role of Wnt signalling in the epiblast of mammalian embyos (see for example Davidson et al., PNAS 2012 and Blauwkamp Nat Commun 2014). I would strongly recommend to delete the last phrase of the paragraph as the Wnt-based parallel between CTCs and hESC appears extremely weak and fails to acknowledge the vast literature of the hESC field on this topic.
  Authors’ response: *We agree with the Reviewer in that the role of Wnt in hESC is controversial. Accordingly, we have removed the last sentence of the paragraph*.
2. On my point 8. I fear there was a partial misunderstanding. The authors draw a parallel between the culture conditions used for CTCs with those used for hESC, and use this to strenghten the notion that CTCs display some stem cell properties. My warning was on the fact that hESC themselves are not all the same, and do require different culture conditions. The issue is not on mouse vs. human stem cells, but on the fact that two types of mammalian pluripotent cells exist: naive pluripotent cells, like the usual mESC but also like the ground-state hPSC recently derived (which, for example, both require constitutive Wnt activation for self-renewal), and primed pluripotent cells, like mouse Epiblast-SC or the usual hESC (which instead require FGF and TGFbeta stimulation, and differentiate upon Wnt activation). These populations are very similar (much more similar than hESC and CTCs), yet they display opposite or anyway different culture conditions (see Weinberger NRMCB 2016). Therefore, it appears arbitrary to establish a parallel with only one human pluripotent stem cell population only because this one fits. However, I respect the author’s choice to keep their idea.
  Authors’ response: *We thank the Reviewer for his comment. However, we clearly point out in the manuscript that the parallel between culture conditions for CTCs and hESCs is speculative, yet not conclusive about their identity*.

### Original comments

Summary: The review starts from the very interesting finding that circulating tumor cells (CTC) can be found not only as single cells but also in small clusters, both in mouse models for metastatic cancer and in human patients suffering from solid tumors. Moreover, the authors found that CTC clusters display higher tumor-seeding ability in mice, and that their presence in the blood of human patients is highly predictive of disease relapse and metastatic disease. The authors discuss their findings in light of the notion that metastasis is the actual cause of death form many cancer patients, thus calling for a better understanding of how cells form metastasis, and also discuss the possible links between CTC biology and other traits that have been previously associated with metastatic propensity, such as EMT and stem-like properties. The topic is very up-to-date and interesting, as it opens a series of challenging and partially unanswered questions.

Authors’ response: *We thank the Reviewer for his favorable comments on our manuscript.*

Recommendations:

1. In several points of the review the authors discuss their findings in light of current mainstream theories about metastasis. For the sake of the non-expert audience, I think it would be valuable to sometimes briefly introduce these theories in a more balanced way, by providing the reader also with the possible pitfalls of a given idea/theory, mostly if these pitfalls are based on experimental evidence. In my idea, a review should not only serve to put a paper in the mainstream, but also to highlight what we still don’t know or don’t fully understand in a field, and which should be a priority for future studies.
  Authors’ response: *We fully agree with the point of view of the Reviewer. In our review, we attempted to provide a wide overview of the opportunities as well as the limitations of the CTC field, with a focus on those assays used for their isolation and characterization, and a special attention to describe what is currently known in regard to their stem-like properties. We also provide a number of speculations and hypotheses that may be of interest to a broad readership*.
2. Page 1, around line 45. In the discussion of the correlation between CTC and metastasis in human patients, it would be interesting to know whether prospective studies have been carried out to causally link the presence/abundance of CTCc and the prognosis, above correlative studies. If not yet available, this review could be the right place to recommend such important type of studies.
  Authors’ response: *A high number of prospective studies have been conducted for CTCs in several cancer types, including breast, colorectal and lung cancer (e.g. see Li et al., Oncotarget, 2016; Bork et al., Br J Cancer, 2015; Lucci et al., Lancet Oncology, 2012; Mego et al., Breast Cancer Research, 2015; Wallwiener et al., BMC Cancer, 2014; Rack et al., JNCI, 2014). These studies clearly pointed to a high prospective value of CTCs in predicting disease progression or response to therapy. In this review, we therefore decided to not point out the need for additional prospective studies*.
3. Page 5: was the correlation between cluster CTC and malignancy observed in human cancer patients corrected for the grade/burden of the primary tumor? In other words, are cluster CTC observed only in patients with bigger/more advanced tumors? If so, maybe the presence of CTC is only a readout of the tumor malignancy, rather than a cause of recurrence per sé?
  Authors’ response: *These studies focused on patients with a progressing metastatic disease, all generally characterized by a high tumor burden. No direct correlation between the presence of CTC clusters and tumor grade or burden was found. We have now updated the text, where we clarify that a correlation between CTC clusters and poor prognosis was evident in patients with a metastatic disease.*
4. Page 5: What can we learn, or at least estimate, on human disease from the CTC half-life in mice and the observed CTC frequencies in human patients? Experiments in mice were based on the i.v. injection of many cells (2*10^5), which is perhaps very high compared to the real situation in humans. Still, the authors easily tracked single-cell and cluster CTCs in human samples. It would be interesting to estimate the number of CTC released in the blood by human tumors, as a tentative measure of the frequency and importance of the phenomenon.
  Authors’ response: *We agree with the Reviewer that it would be extremely interesting to estimate the number of CTCs that are directly released in circulation from a human tumor lesion. Especially, it would be interesting to assess how many of them, and how quickly, are entrapped within small capillaries during their first pass through the circulatory system. Given the short half-life of CTCs in mouse models, we believe that such studies would be exceptionally challenging in patient samples. In our review, we dedicate a full paragraph discussing the implications of a short circulation half-life of CTCs. Clearly, more studies will be needed to better estimate and describe this process in cancer patients.*
5. Page 6: It should be noted and discussed here that the concept of CSC, although an interesting one, is not universally accepted and is for sure greatly biased by the experimental system: it is indeed widely known that the estimated CSC (or tumor-seeding) frequency in cancer cell populations can widely differ from small percentages to basically 100 % of cells depending on the mouse strain and on the conditions in which the assay is carried out. Unless these studies have been revised and updated recently, this should be incorporated in the discussion as a precautionary information, because this suggests the possibility that the “tumor initiating cell” phenotype, on which the definition of a CSC marker is often based, reflects cell populations with increased proliferation or survival ability, rather than true “stem cells”.
  Authors’ response: *With this comment, the Reviewer raises a very important point. We have addressed this comment in two parts. First, we comment that using different recipient mouse strains but also assaying tumors at different stages of progression may yield significant differences in the putative cancer stem cell population. Second, we note that the functional assays currently used are not refined enough to distinguish bona fide cancer stem cells from cells with increased proliferation or survival ability, since they interrogate these properties at a population level as opposed to the single cell level.*
6. Page 8 The finding that hypoxia needs to be used in vitro to culture CTCs is not expected, but something strange, because CTC are exposed to the highest oxygen concentrations into the bloodstream. This should be mentioned and discussed also because hypoxia changes a wide number of parameters above and beyond the activity of HIFs, including gene expression and metabolism, which might be not so relevant to mimic in vitro the vivo situation.
  Authors’ response: *We have now expanded the paragraph that discusses CTC culture in hypoxic conditions, to comment on this specific point. For what concerns oxygen levels in circulation, we would like to point out that these are known to vary dramatically and rapidly, from arterial to venous blood. For example, venous blood is thought to have a much lower oxygen concentration than the intestinal tissue or the kidney. Given the short half-life of CTCs in circulation, the need for hypoxic conditions could rather reflect that these cancer cells were adapted to a hypoxic environment within their tumor tissue of origin. We provide now a better description of this phenomenon within the text, and we thank the Reviewer for highlighting this aspect.*
7. Indeed the argument itself that a culture condition (e.g. hypoxia or FGF) suitable for hESC can teach us something significant on other stem cells, including cancer stem cells, is very simplistic, and tends to draw the idea that stem cells are all the same. Just to make an example, hESC and mESC, despite a very high degree of similarity, have COMPLETELY different culture conditions, such that one type does not survive in the conditions suitable for the other (and in particular in the presence of FGF). This last part should be carefully reconsidered.
  Authors’ response: *In this particular case we respectfully disagree with the comment of the Reviewer. When considering human cells, these culture conditions seem to strongly favor the propagation of stem-like cells in multiple systems, therefore we still believe that it is important to speculate on this observation in the context of this review. With this in mind, we are certainly not arguing that stem cells are all the same. In fact, we agree that mouse ESCs require very different propagation conditions, and we have now pointed out this aspect in the text. The updated text should now provide more clarity and a better background concerning propagation conditions for stem-like cells.*

Minor issues:

1. Page 4, around line 12 The problem of counter-selection against CTCs displaying mesenchymal traits should be better explained in light of the findings that in some solid tumors the most aggressive cells (in terms of metastatic potential and cancer-stem cell traits) are thought to be the ones undergoing EMT. This is of course still a matter of debate (i.e. the fact that EMT is truly observed in tumors, and instrumental for metastasis) and requires a bit more of explanation.
  Authors’ response: *We thank the reviewer for his comment. We have better described the concept of EMT later in the paper, when discussing stem-like properties of cancer cells in circulation.*
2. Page 6, around line 55 Here the authors mix two different concepts: one thing is the identification of bona fide universal CSC markers in a given tumor type; another is the cell of origin of tumors. That tumors can form when mice are manipulated to express an oncogene into a physiologic stem cell compartment, such as LGR5+ intestinal stem cells, is not so surprising, and not so informative on the actual cell of origin of cancer (by definition, the activated oncogene will be present in the stem cell but also in all its progeny). The real and often unanswered question is whether oncogenic mutations in normal differentiated cells can reprogram those cells to a stem-cell phenotype, which at least in part recapitulates traits of the physiological stem cells of the tissue of origin. Please also remember that LGR5 was isolated as a normal stem cell marker based on the hypothetical assumption that cancer cells recapitulate traits of the physiologic stem cell (VanDeWetering and Clevers original paper), but how many of the physiological stem cell markers identified thereafter do follow this rule and are also found in cancer stem cell populations?
  Authors’ response: *We have rephrased this part of the text according to the Reviewer’s suggestion. For colon and ovarian tumors we indicate that the use of the bona fide cancer stem cell marker CD133 enriches for cancer-initiating cells. We have deleted the reference to LGR5+, as the Reviewer is correct and it may generate confusion in regard to the concepts of cell of origin versus cancer stem cell.*
3. Page 7, after line 30 The idea that metastatic cells are endowed with stem-like properties is not entirely new and should be referenced.
  Authors’ response: *We agree with the Reviewer and have changed the text accordingly.*
4. It seems to me however that there is a logical gap between what the authors originally observed in Aceto et al., Cell 2014, and the link between CTC and CSC phenotypes. The authors originally used a clonal cell line such as MDA-LM2, and there is to my knowledge poor evidence that these cells can be fractionated into stem-cells and non-stem-cells (i.e. differentiated). To which extent a homogeneous system like MDA-LM2 is informative on heterogeneous cancer populations containing both stem and non-stem cancer cells?
  Authors’ response: *We respectfully disagree with the Reviewer in this case. MDA-MB-231 cells have been originally derived from a pleural effusion of a Caucasian breast cancer patient, and they are not considered a clonal cell population. MDA-MB-231 cells have also been shown by several groups to contain a stem-like subpopulation, usually referred to as the CD44*^*+*^*/CD24*^*-*^*subpopulation, with enhanced invasive and tumorigenic properties, as well as a population of non stem-like cells.*
5. Page 9, around line 28 The term “pluripotency” is usually restricted to very early embryonic stem cell populations. The papers referenced here show the capacity of one committed mammary precursor population to revert to a more primitive state and differentiate into other mammary lineages into which it would not differentiate in the physiologic state. Please use the term “multipotency” as in the title of these papers.
  Authors’ response: *We agree with the Reviewer and have accordingly replaced the term pluripotency for the term multipotency within the text.*
6. Page 9, around line 30 It is not clear why a low frequency mutation should be particularly interesting: the tenet is that a mutation is retained proportionally to the selective advantage it provides to cancer cells, and acquisition of stem-like features should be an advantage for the primary tumor, not only for CTCs or for metastasis.
  Authors’ response: *We agree with the comment of the reviewer. However, it is important to mention that the mutational profile in patients is dynamic and it evolves as a function of time and resistance to therapy. Low-frequency mutations that may confer stem-like properties and are detected in a minority of cells at a certain point should not be overlooked, since they may become prevalent during disease progression. We have adapted the text to better describe this concept*.
7. Finally, something that is completely missing and may merit some discussion is how CTC clusters are formed in the first instance, as opposed to single-cell CTC, and how do they extravasate. Much work has been done in recent years on the ability of cancer cells to undergo collective cell migration instead of the more classical amoeboid single-cell migration. However, this was used in some cases to advocate against the need of EMT in solid tumors (EMT would facilitate the detachment of single cells from the epithelial mass and reprogram the cell to a migratory behavior). How then these different aspects would merge in the case of CTC clusters?
  Authors’ response: *We agree with the Reviewer that the processes of CTC intravasation and extravasation are of great importance. While we feel that the intravasation processes (e.g. via collective versus single cell migration) are beyond the scope of this review, we have now mentioned two studies that highlight possible extravasation mechanisms of CTCs, mostly in the context of their cellular heterogeneity, i.e. their interaction with platelets.*

## Abbreviations

ALDH, alcohol dehydrogenase; AML, acute myeloid leukemia; CD, cluster of differentiation; CK, cytokeratin; CTCs, circulating tumor cells; CTLA-4, cytotoxic T lymphocyte antigen-4; CXCR4, C-X-C Chemokine Receptor Type-4; DAPI, 4’,6-diamidino-2-phenylindole; EGF, epidermal growth factor; EMT, epithelial-to-mesenchymal transition; EpCAM, Epithelial Cell Adhesion Molecule; ESA, epithelial-specific antigen; FBF2, basic fibroblast growth factor; hESCs, human embryonic stem cells; hIPSCs, human induced pluripotent cells; JAK, janus kinase; KLF4, kruppel-like factor 4; LGR5, leucine-rich repeat-containing G-protein coupled receptor 5; MAPK, mitogen-activated protein kinase; PD1, programmed death 1; PD-L1, programmed death ligand 1; PI3K, phosphatidylinositol 3-kinase; PTEN, phosphatase and tensin homolog; RBCs, red blood cells; STAT, signal transducer and activator of transcription; TGF-β, transforming growth factor-β; WBCs, white blood cells; WNT, wingless-type MMTV integration site
